# The rock damage mechanism of combined rock breaking with saw blade and conical pick

**DOI:** 10.1038/s41598-023-46442-z

**Published:** 2023-11-11

**Authors:** Zhiwen Wang, Qingliang Zeng, Lirong Wan, Zhenguo Lu, Hongxiang Jiang, Saurabh Dewangan

**Affiliations:** 1 College of Transportation, Shandong University of Science and Technology, Qingdao, 266590 China; 2 College of Mechanical and Electronic Engineering, Shandong University of Science and Technology, Qingdao, 266590 China; 3 College of Information Science and Engineering, Shandong Normal University, Jinan, 250358 China; 4 College of Mechatronic Engineering, China University of Mining and Technology, Xuzhou, 221116 China; 5 Department of Mechanical Engineering, Manipal University Jaipur, Jaipur, Rajasthan 303007 India

**Keywords:** Civil engineering, Fossil fuels

## Abstract

The combined rock breaking method with the saw blade and conical pick is proposed to improve the rock breaking efficiency. The numerical simulation of combined rock breaking with the saw blade and conical pick is established to investigate the rock damage mechanism. And verified and modified the numerical simulation model with the rock breaking comprehensive test bench, the quantitative analysis error is less than 0.05, indicated quantitative analysis system is accuracy. The result indicated that the cutting parameters of the saw blade and conical pick affect the rock damage. And the cutting parameters of conical pick and structural parameters of rock plate have been studied to influence rock breaking volume. The research result could help optimize the cutting parameters of the saw blade and conical pick to improve the rock breaking efficiency.

## Introduction

With the rapid development of tunneling technology, rock breaking technology is widely used in roadway tunneling, mainly including drilling and blasting rock breaking, mechanical rock breaking, thermal rock breaking and high-pressure water jet rock breaking methods. However, drilling, blasting and mechanical rock breaking technology are relatively mature now, while other rock breaking methods are still in the research stage and have not been widely applied. The drilling and blasting and mechanical rock breaking methods can peel off rock through the external force to realize the roadway excavation.

In the engineering application, however, the method of increasing the tunneling equipment's ability to rock breaking is often adopted to improve the tunneling equipment's ability with increasing installed power. Which caused the increased wear of the cutting mechanism, increased dust volume of the working flour, and deterioration of the working face environment. Which has no significant effect on improving the tunneling capacity and speed of the mining equipment, and there are large potential safety hazards. Therefore, to reduce the tunneling equipment's volume and weight, improve the tunneling equipment's adaptability to the hard rock crushing working environment, and have a high cutting capacity and efficiency. It is urgent to study a new rock breaking method, and then research and develop advanced tunneling equipment, accelerate the construction speed of rock roadway tunneling, and ensure normal tunneling. In the paper, the method of combined rock breaking with the saw blade and conical pick is proposed to improve the rock breaking ability of the tunneling equipment. The saw blade is applied in the combined rock breaking method to increase rock surface number, which could reduce the rock strength, and then the conical pick breaks the rock with saw blade pre-cutting. The combined rock broken method could improve the rock breaking efficiency and ability. Which is of great significance for efficient tunneling.

Many scholars at home and abroad have done much research on rock fragmentation with simulation and experiment methods. Li et al.^1^ simulated the influence of confining pressure on the rock fracture pressure of conical pick with the discrete element method (DEM), and analyzed the rock fracture process, cutting force and specific cutting energy of four kinds of rock under different confining pressures. Wang et al.^2^ pointed out that rock breaking performance of conical pick is affected by the confining pressure, and proved that the brittleness under biaxial confining pressure differs from that under uniaxial confining pressure through experiments and regression analysis. The rock is more fragile under uniaxial confining pressure and more fragile when confining pressure is released. Jiang et al.^3^ studied the influence of different water jet rock breaking cutting heads on energy consumption, compared the influence of water jet on rock breaking performance, and determined the best parameters of water jet-assisted rock breaking.

The cutting parameters and rock material parameters effect on cutting performance have been studied with experiment and method. Yasar et al.^4^ conducted rock cutting experiments with rock cutting machine cutting six kinds rock to research influence of cutting depths and linear cutting speeds on cutting force and specific cutting energy. Wang et al.^5^ conducted indentation tests on rock broken by conical picks based on rock fracture mechanics and combined with Evan's^6–8^ rock breaking theory to study the factor of rock broken by conical pick. Zou et al.^9^ investigated the rock fragments in the process of rock breaking based on PFC(2D), and pointed the cutting depths and cutting angles greatly influence average cutting force.

Many scholars investigated conical pick cutting breaking rock process with numerical simulation method. Liu et al.^10^ established a numerical simulation model of rock cutting with conical pick based on using the discrete element method and studied the cutting speed, cutting angle and installation angle of conical pick on the cutting performance. Wan et al.^11^ proposed a method to judge the cutting performance of conical pick on random load, and the numerical simulation results indicated that the number of conical picks, installation angle and cutting position have gradually reduced the impact on the cutting performance of cylinder. Fan et al.^12^ simulated conical pick breaking rock based on 3D SPH simulation model. Zhou et al.^13, 14^ studied the influence of conical pick angle, cutting angle and cutting speed on rock crushing dust, indicating that increasing conical pick angle and cutting angle of the conical pick is conducive to reducing dust generation. Che et al.^15^ conduced a series of linear cutting tests to observe the process of fragment formation. Goktan et al.^16^ proposed an improved theory considering the friction angle and compressive strength of rock, in addition, a large number of simulations were conducted on rock cutting to study fragment separation and obtain scientific results^17–26^. Lu et al.^27^ studied the rock plate structural parameters influence on peak cutting force of conical pick based on saw blade pre-cutting.

The saw blade cutting performance has been investigated with the numerical simulation and experiment and theory methods. Karakurt et al.^28^ and Sengun et al.^29^ studied the cutting force and specific cutting energy in the saw blade cutting rock process. Hellstrom et al.^30^ study the saw blade cutting rock process with theory and experiment methods. Yasitli et al.^31^ established the discrete element model to study the tangential speed and feed speed effect on cutting performance. Xu et al.^32, 33^ studied the cutting force and specific cutting energy of saw blade cutting rock, and studied the force ratio by experiment method. Using numerical simulation, Zeng et al.^34^ studied the cutting force of saw blade and rock damage with different cutting parameters. Nasir et al.^35, 36^ applied statistical regression and artificial intelligence method to correlate AE-extracted features with sawing capacity and waviness to predict cutting power and waviness and the impact of cutting parameters on cutting energy consumption. Turchetta et al.^37^ evaluated the cutting performance of the saw blade by measuring the cutting force and wear, and analyzed the impact of rock physical characteristics, cutting parameters, saw blade structure parameters, etc. on the production efficiency. Bilek et al.^38^ analyzed the influence of the cutting speed, cutting force, installation and shape of the saw blade with the finite element method, and analyzed the key reasons for the damage of the saw blade. Chen et al.^39^ established a sawing model based on a finite element to study the cutting parameters. Based on the load, effective stress, and cutting temperature, good blade wear, they fully considered the interaction of cutting parameters and optimize the structural design.

Scholars at home and abroad have done a lot of research on rock fragmentation, mainly focusing on the impact of cutting parameters and rock property parameters on the cutting performance and tool wear performance of the pick. However, the research on rock damage in rock breaking is less and lacks the necessary quantitative analysis method. The quantitative analysis method is proposed to study the influence of cutting parameters and rock property on rock damage. The rock damage quantitative research is achieved, through a rock damage quantitative analysis is established based on image recognition. To improve the tunneling efficiency of hard rock tunnel, this paper proposes a combined rock breaking method of saw blade and pick to improve the rock breaking ability of tunneling equipment. In the process of combined rock breaking by saw blade and pick, the cutting parameters of the tool and the rock structure parameters of the multi free surface formed by the pre-cutting of the rock by the saw blade affect the rock damage and the rock breaking volume. Set up a comprehensive test bench for rock breaking by saw blade and pick, conduct rock breaking test by saw blade and pick, verify and verify the numerical model. And systematically study the relationship between rock damage and breaking and cutting parameters of the cutter and structural parameters of rock plate formed by pre-cutting of the saw blade and pick in the process of rock breaking by saw blade and pick through numerical simulation. The research results will lay a foundation for promoting rock breaking by saw blade and pick.

## Methods

### Rock damage theory

**Figure 1:**
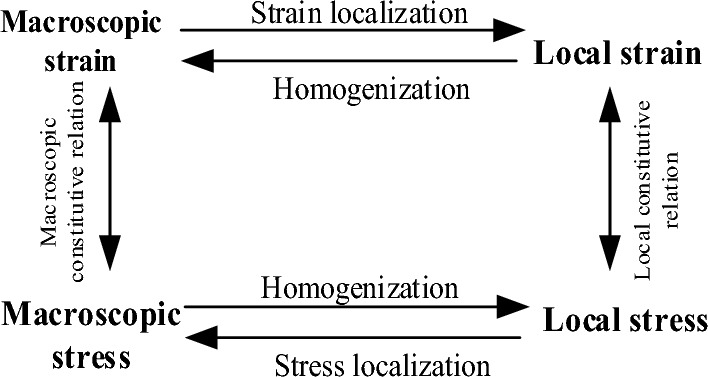
The research way of homogenization.

According to the rock's heterogeneity and porosity, the rock's damage mechanism is studied. The mechanism of rock damage evolution is studied based on the theory of rock damage caused by crack propagation of heterogeneous material homogenization mechanical properties and thermodynamic theory. To study the mechanical behavior of rock materials, the homogenization theory is adopted. The homogenization method generally adopts research path shown in Fig. 1.

Assume that there is a macroscopic uniform stress field at the outer boundary $\partial {\text{A}}$ of a specific element A as $\overline{\sigma }$, that is, equivalent to an additional uniform surface load ${\text{t}}^{{1}} \left( {\text{x}} \right){ = }\overline{\sigma } \cdot {\text{n}}\left( {\text{x}} \right){\text{, x}} \in \partial {\text{A}}$. The internal stress field of the characteristic element satisfies the boundary conditions as $\sigma_{{\text{x}}} \cdot {\text{n}}\left( {\text{x}} \right){ = }\overline{\sigma } \cdot {\text{n}}\left( {\text{x}} \right){\text{, x}} \in \partial {\text{A}}$. When the physical force is ignored, the average local stress on the characteristic element body and the boundary stress satisfies Eq. (1).

1 $$\sigma_{{\text{A}}} = \frac{1}{{\text{V}}}\mathop \smallint \limits_{{\text{A}}}^{{}} \sigma ({\text{x}}){\text{dv}} = \overline{\sigma }$$

among that, *V*-volume of feature element, ${\langle \sigma \rangle }_{A}$-average value on a characteristic element. Leave out the characteristic element body by *A* and the static equilibrium condition at any point in the characteristic element $div\sigma ({\text{x}}) = 0$. Combined relation equation $\partial x_{i} /\partial x_{j} = \delta_{ij}$, and the stress equation is shown in Eq. (2).

2 $$\sigma_{{{\text{ij}}}} = \sigma_{{{\text{ik}}}} \delta_{{{\text{ik}}}} = \sigma_{{{\text{ik}}}} \frac{{\partial {\text{x}}_{{\text{i}}} }}{{\partial {\text{x}}_{{\text{k}}} }} = \left( {\sigma_{{{\text{ik}}}} {\text{x}}_{{\text{j}}} } \right)_{{\text{,k}}} - \sigma_{{{\text{ik}},{\text{k}}}} {\text{x}}_{{\text{j}}} = \left( {\sigma_{{{\text{ik}}}} {\text{x}}_{{\text{j}}} } \right)_{{,{\text{k}}}}$$

The relationship equation is established based on the Gaussian theorem, as shown if Eq. (3). The average value of the macroscopic uniform stress acting on the external boundary of the element is equal to that of the local stress in the characteristic element.

3 $$\int\limits_{{\text{A}}} {\sigma_{{{\text{ij}}}} \left( {\text{x}} \right){\text{dV}} = {\text{V}}\overline{\sigma }_{{{\text{ik}}}} \delta_{{{\text{jk}}}} = {\text{V}}\overline{\sigma }_{{{\text{ij}}}} }$$

Local strain under small deformation condition *ε*(*x*) of the average volume strain on the characteristic element is equal to $\overline{\varepsilon }$, shown as Eq. (4).

4 $$\varepsilon {\text{(x) = }}\frac{{1}}{{\text{V}}}\int\limits_{{\text{V}}} {\varepsilon {\text{(x)dV = }}\overline{\varepsilon }}$$

At the same time, under the condition of small deformation can be obtained by combining the Gaussian theorem, shown as Eq. (5).

5 $$\int\limits_{{\text{V}}} {\varepsilon {\text{(x)dV}}} { = }\frac{{1}}{{2}}\int\limits_{{\partial {\text{A}}}} {\left( {{\text{u}}_{{\text{i}}} {\text{n}}_{{\text{j}}} {\text{ + u}}_{{\text{j}}} {\text{n}}_{{\text{i}}} } \right){\text{dS}} = \frac{{\text{V}}}{{2}}\left( {\overline{\varepsilon }_{{{\text{ij}}}} { + }\overline{\varepsilon }_{{{\text{ji}}}} } \right)}$$

Assume that all material phases in the characteristic elements are linear elastic mechanical behavior and conform to the generalized Hook's law that is $\sigma \left(x\right)={\mathbb{C}}\left(x\right):\mathcal{E}\left(x\right), x\in A$. ${\mathbb{C}}\left(x\right)$-local elastic tensor. The elastic tensor of rock mass (Phase 1#) is ${\mathbb{C}}^{0}$, and that of impurity phase (r + 1), and that of impurity phase (r + 1) is ${\mathbb{C}}^{c,r}$, r = 0, 1, 2,……., N. The macroscopic mechanical properties of solid matrix impurity systems are linear elastic.

They correlate the local strain tensor with the macro strain to obtain the equation $\langle \varepsilon (x)\rangle =\langle {\mathbb{A}}\left(x\right):\overline{\varepsilon }\rangle =\langle {\mathbb{A}}\left(x\right)\rangle :\overline{\varepsilon }$. Owing to $\langle \varepsilon (x)\rangle =\overline{\varepsilon }$, and the strain localization tensor properties $\langle {\mathbb{A}}\left(x\right)\rangle ={\mathbb{I}}$.

Among that, ${\mathbb{I}}$ is the fourth order symmetric element tensor, based on the second order symmetric element tensor $\delta$. The tensor ${\mathbb{I}}$ is shown as Eq. (6).

6 $${I}_{ijkl}=\frac{1}{2}\left({\delta }_{ik}{\delta }_{jl}+{\delta }_{il}{\delta }_{jk}\right)$$

The homogenization result of the stress tensor on the characteristic element body is $\tilde{\sigma }=\langle \sigma \left(x\right)\rangle =\langle {\mathbb{C}}\left(x\right):{\mathbb{A}}\left(x\right):\overline{\varepsilon }\rangle =\langle {\mathbb{C}}\left(x\right):{\mathbb{A}}\left(x\right)\rangle :\overline{\varepsilon }$. And then the effective tensor ${\mathbb{C}}^{hom}$ expression for heterogeneous material is ${\mathbb{C}}^{hom}=\langle {\mathbb{C}}\left(x\right):{\mathbb{A}}\left(x\right)\rangle$. At the same time, the macro stress–strain relationship is $\tilde{\sigma }={\mathbb{C}}^{hom}:\overline{\varepsilon }$.

Under the external load, the random distribution of cracks in the characteristic element body causes the rock to show significant anisotropy. Considering the discreteness of the normal vector of rock cracks, that is, the damage variable describing the state of cracks. The damage variable composition set of cracks $C=\left\{{c}_{1},{c}_{2},{c}_{3},{c}_{4},\dots \dots ,{c}_{N}\right\}$, ${c}_{i}$ indicates the *i*-th crack damage value.

Assuming the normal vector ${\varvec{n}}$ of crack, and $d=d\left({\varvec{n}}\right)$ is used to represent the distribution density of rock fractures, and the free energy expression of the fracture matrix system is determined, according to the thermodynamic principle, to establish the damage mechanics, shown as Eq. (7).

7 $$\psi \left( {\varepsilon ,\;d} \right) = \frac{1}{2}\varepsilon :{\mathbb{C}}^{hom} \left( d \right):\varepsilon$$

Among that, ${\mathbb{C}}^{hom}\left(d\right)$-effective elastic tensor of damaged materials.

The state variable is the thermodynamic force associated with the internal variable, and there is free energy to derive the internal variable energy to obtain the energy dissipation of the system. Established the macro stress–strain relationship as shown in Eq. (8).

8 $$\sigma =\frac{\partial \psi }{\partial d}={\mathbb{C}}^{hom}\left(d\right):\varepsilon$$

Obtain the thermodynamic force and damage driving force associated with the damage variable, shown as ${F}_{d}=-(\partial \psi /\partial d)=-(\varepsilon /2):({\partial {\mathbb{C}}}^{hom}(d):\varepsilon$.

The effective mechanical properties of materials are determined through micromechanics, and the mechanical mechanism and mechanism of material damage and failure need be fully considered. The constitutive equation and internal variable evolution criterion are established. According to the second law of thermodynamics, the energy dissipation caused by a crack expansion is nonnegative, which is meeting ${D}_{e}={F}_{d}\dot{d}\ge 0$.

On the basis of thermodynamics, the damage criterion based on strain energy release rate is adopted $g\left({F}_{d},d\right)={F}_{d}-R\left(d\right)\le 0$. Among that, $R\left(d\right)$-resistance function of damage evolution (crack propagation). For the damage criterion, conditions are as shown in Eq. (9)

9 $$\left\{\begin{array}{cc}{F}_{d}=R\left(d\right),\dot{d}>0& \mathrm{Fissure extnsion}\\ {F}_{d}<R\left(d\right),\dot{d}=0& \mathrm{Crack without expansion}\end{array}\right.$$

The need for analytical mathematics expression and the convenience of numerical program research are considered in micro damage mechanics research. It is assumed that the crack propagation process is self similar and only expands in its own plane, so the normal vector of the crack surface does not change. The rock is assumed to be a normal orthogonal material, and the damage evolution follows the orthogonalization criterion.

10 $$\dot{d}={\lambda }^{d}\frac{\partial g\left({F}_{d},d\right)}{\partial {F}_{d}}={\lambda }^{d}, {\lambda }^{d}\ge 0$$

In which, ${\lambda }^{d}$-damage multiplier. The damage evolution equation considering loading and unloading conditions is shown in Eq. (11).

11 $$\dot{d}=\left\{\begin{array}{cc}0& g<0 or g=0 and \dot{g}<0\\ {\lambda }^{d}& g=0 and \dot{g}=0\end{array}\right.$$

The damage multiplier ${\lambda }^{d}$ The damage consistency condition is determined as Eq. (12).

12 $$\dot{g}=\frac{\partial g}{\partial \varepsilon }:\dot{\varepsilon }+\frac{\partial g}{\partial d}\dot{d}=0$$

Based on the damage evolution criterion, the stress–strain relationship in the form of rate is established. The macro stress–strain relationship is expressed in a differential form $\dot{\sigma }={\dot{\mathbb{C}}}^{hom}:\upvarepsilon +{\mathbb{C}}^{hom}:\dot{\varepsilon }$, and the relationship equation is shown as (13).

13 $${\dot{\mathbb{C}}}^{hom}:\upvarepsilon =\frac{\partial {\mathbb{C}}^{hom}:\varepsilon }{\partial d}\dot{d}=\frac{\partial \psi }{\partial \varepsilon \partial d}\dot{d}=-\frac{\partial {F}_{d}}{\partial \varepsilon }{\lambda }^{d}=-\frac{\partial g}{\partial \varepsilon }{\lambda }^{d}$$

That is, $\dot{\sigma }={\mathbb{C}}^{tan}:\dot{\varepsilon }$, among that ${\mathbb{C}}^{tan}$-tangent elastic tensor, the specific expression is shown as ${\mathbb{C}}^{tan}={\mathbb{C}}^{hom}-\partial g/({H}_{d}\partial \varepsilon )\otimes \partial g/\partial \varepsilon$, among that, the damage hardening parameter ${\text{H}}_{{\text{d}}} = - \partial {\text{g/}}\partial \varepsilon$.

### Constitutive model of rock material

The paper selects the RHT material as the rock material model, with the constitutive model tests of different rock materials. The constitutive model of RHT material, coupling shear force and pressure. The relation describes the pressure by polynomial Hugoniot curve and p-α form of compaction. For the compression model, define a variable whose initial value is greater than 0 representing porosity, which represents the material's density fraction and decreases with the increase of pressure. The reference density can be expressed as *αρ*. The porosity is shown in Eq. (14).

14 $$\alpha \left( {\text{t}} \right){ = }\max \left( {{1},\min \left\{ {\alpha_{{0}} ,\min_{{{\text{s}} \le {\text{t}}}} \left[ {{1 + }\left( {\alpha_{0} - 1} \right)\left( {\frac{{{\text{p}}_{{{\text{comp}}}} - {\text{p(s)}}}}{{{\text{p}}_{{{\text{comp}}}} - {\text{p}}_{{{\text{el}}}} }}} \right)^{{\text{N}}} } \right]} \right\}} \right)$$

Among that, $\alpha$(*t*)-the pressure of time *t*, *p*_*el*_-initial pore crushing pressure, *p*_*comp*_-compaction pressure, and *N*-porosity index. To define the cap pressure, the current pore crushing pressure equation is as follows Eq. (15)

15 $${\text{p}}_{{\text{c}}} {\text{ = p}}_{{{\text{comp}}}} - \left( {{\text{p}}_{{{\text{comp}}}} - {\text{p}}_{{{\text{el}}}} } \right)\left( {\frac{\alpha - 1}{{\alpha_{0} - 1}}} \right)^{{{\raise0.7ex\hbox{${1}$} \!\mathord{\left/ {\vphantom {{1} {\text{N}}}}\right.\kern-0pt} \!\lower0.7ex\hbox{${\text{N}}$}}}}$$

The residual pressure model is determined by the porous density ρ and the specific internal energy *e*. According to the input value, it is determined by *B*_*0*_ (${B}_{0}>0$).

16 $$p\left( {\rho {\text{,e}}} \right){ = }\frac{{1}}{\partial }\left\{ {\begin{array}{*{20}c} {\left( {{\text{B}}_{{0}} {\text{ + B}}_{{1}} } \right)\partial \rho {\text{e + A}}_{{1}} \eta {\text{ + A}}_{{2}} \eta^{{2}} {\text{ + A}}_{{3}} \eta^{{3}} } & {\eta > 0} \\ {{\text{B}}_{{0}} \partial \rho {\text{e + T}}_{{1}} \eta {\text{ + T}}_{{2}} \eta^{{2}} } & {\eta < 0} \\ \end{array} } \right.$$

When *B*_*0*_ = 0, *p* $\left( {\rho {\text{,e}}} \right){ = }\Gamma \rho {\text{e + }}\frac{{1}}{\partial }{\text{p}}_{{\text{H}}} \left( \eta \right)\left( {{1} - \frac{{1}}{{2}}\Gamma \eta } \right)$, ${\text{p}}_{{\text{H}}} \left( \eta \right){\text{ = A}}_{{1}} \eta {\text{ + A}}_{{2}} \eta^{{2}} {\text{ + A}}_{{3}} \eta^{{3}}$, and *η* $\left( \rho \right) = \partial \rho /(\partial_{0} \rho_{0} ) - 1$, shear strength ${\text{p}}^{*}\text{=p/}{\text{f}}_{\text{c}}$. s-deviatoric stress tensor, ${\dot{\varepsilon }}_{p}$-plastic strain rate. ${\varepsilon }_{p}^{*}$-effective plastic strain. For a given stress state and loading rate, the elastic–plastic yield surface of the RHT model is ${\sigma }_{y}\left({p}^{*}, s, {\dot{\varepsilon }}_{p},{\varepsilon }_{p}^{*}\right)={f}_{c}{\sigma }_{y}^{*}\left({p}^{*},{F}_{r}\left({\dot{\varepsilon }}_{p},{p}^{*}\right),{\varepsilon }_{p}^{*}\right){R}_{3}\left(\theta ,{p}^{*}\right)$

Combine the two functions and the compression strength parameter ${f}_{c}$. The first describes the principal stress condition pressure relationship ${\sigma }_{1}<{\sigma }_{2}={\sigma }_{3}$. The failure surface and normalized plastic strain ${\sigma }_{y}\left({p}^{*},{F}_{r},{\varepsilon }_{p}^{*}\right)={\sigma }_{f}^{*}\left({p}^{*}/\gamma ,{F}_{r}\right)\gamma$.

At the same time, $\gamma ={\varepsilon }_{p}^{*}+\left(1-{\varepsilon }_{p}^{*}\right){F}_{e}{F}_{c}$, the relationship equation is shown as Eq. (17)

17 $$\sigma _{f}^{*} \left( {p^{*} ,F_{r} } \right) = \left\{ {\begin{array}{*{20}c} {\begin{array}{*{20}c} {A\left[ {p^{*} - \frac{{F_{r} }}{3} + \left( {\frac{A}{{F_{r} }}} \right)^{{ - 1/n}} } \right]^{n} } & {3p^{*} \ge F_{r} } \\ {\frac{{F_{r} f_{s}^{*} }}{{Q_{1} }} + 3p^{*} \left( {1 - \frac{{f_{s}^{*} }}{{Q_{1} }}} \right)} & {F_{r} > 3p^{*} \ge 0} \\ \end{array} } \\ {\begin{array}{*{20}c} {\frac{{F_{r} f_{s}^{*} }}{{Q_{1} }} - 3p^{*} \left( {\frac{1}{{Q_{2} }} - \frac{{f_{s}^{*} }}{{Q_{1} f_{t}^{*} }}} \right)} & {0 > 3p^{*} > 3p_{t}^{*} } \\ 0 & {3p_{t}^{*} > 3p^{*} } \\ \end{array} } \\ \end{array} } \right.$$

Among that, ${p}_{t}^{*}=\frac{{F}_{r}{Q}_{2}{f}_{s}^{*}{f}_{t}^{*}}{3\left({Q}_{1}{f}_{t}^{*}-{Q}_{2}{f}_{s}^{*}\right)}$ is the failure crushing pressure, ${F}_{r}$-dynamic increment factor, and ${Q}_{1}={R}_{3}\left(\pi /6,0\right)$, ${Q}_{2}=Q\left({p}^{*}\right)$。

Use the relative tensile strength ${f}_{t}^{*}$, shear strength ${f}_{s}^{*}$ and compressive strength of concrete ${f}_{c}$ and Q values explain the correlation between tensile and shear meridians. Further details describe the factor that reduces the shear and tensile meridian strength, shown as Eq. (18).

18 $${{R}}_{{3}} \left( {\theta {{,p}}^{*} } \right){ = }\frac{{{2}\left( {{1} - {{Q}}^{{2}} } \right)\it cos \theta { + }\left( {{{2Q}} - {1}} \right)\sqrt {{4}\left( {{1} - {{Q}}^{{2}} } \right){{\it cos}}^{{2}} \theta {{ + 5Q}}^{{2}} - {{4Q}}} }}{{{4}\left( {{1} - {{Q}}^{{2}} } \right){{\it cos}}^{{2}} \theta { + }\left( {{1} - {{2Q}}} \right)^{{2}} }}$$

The relation equation between Load angle $\theta$ and deviator stress tensor *s* is shown in Eq. (19).

19 $$\it cos 3\theta { = }\frac{{{27det(s)}}}{{{2}\overline{\sigma }{{(s)}}^{{3}} }}{,}\overline{\sigma }\left( {{s}} \right){ = }\sqrt {{3}/{{2s:s}}}$$

Relative pressure function reduced the maximum strength as $\text{Q=Q}\text{(}{\text{p}}^{*}\text{)}\text{=}{\text{Q}}_{0}+ \text{B} {\text{p}}^{*}$. And the relative strain rate coefficient is shown as Eqs. (20) and (21)

20 $${\text{F}}_{{\text{r}}} \left( {\dot{\varepsilon }_{{\text{p}}} {\text{,p}}^{*} } \right){ = }\left\{ {\begin{array}{*{20}c} {{\text{F}}_{{\text{r}}}^{{\text{c}}} } & {{\text{3p}}^{*} \ge {\text{F}}_{{\text{r}}}^{{\text{c}}} } \\ {{\text{F}}_{{\text{r}}}^{{\text{c}}} - \frac{{{\text{3p}}^{*} - {\text{F}}_{{\text{r}}}^{{\text{c}}} }}{{{\text{F}}_{{\text{r}}}^{{\text{c}}} {\text{ + F}}_{{\text{r}}}^{{\text{t}}} {\text{f}}_{{\text{t}}}^{*} }}\left( {{\text{F}}_{{\text{r}}}^{{\text{t}}} - {\text{F}}_{{\text{r}}}^{{\text{c}}} } \right)} & {{\text{F}}_{{\text{r}}}^{{\text{c}}} {\text{ > 3p}}^{*} \ge {\text{F}}_{{\text{r}}}^{{\text{t}}} {\text{f}}_{{\text{t}}}^{*} } \\ {{\text{F}}_{{\text{r}}}^{{\text{t}}} } & { - {\text{F}}_{{\text{r}}}^{{\text{t}}} {\text{f}}_{{\text{t}}}^{*} {\text{ > 3p}}^{*} } \\ \end{array} } \right.$$

21 $${\text{F}}_{{\text{r}}}^{{{\raise0.7ex\hbox{${\text{c}}$} \!\mathord{\left/ {\vphantom {{\text{c}} {\text{t}}}}\right.\kern-0pt} \!\lower0.7ex\hbox{${\text{t}}$}}}} { = }\left\{ {\begin{array}{*{20}c} {\left( {\frac{{\dot{\varepsilon }_{{\text{p}}} }}{{\dot{\varepsilon }_{{0}}^{{{\raise0.7ex\hbox{${\text{c}}$} \!\mathord{\left/ {\vphantom {{\text{c}} {\text{t}}}}\right.\kern-0pt} \!\lower0.7ex\hbox{${\text{t}}$}}}} }}} \right)^{{\beta_{{{\raise0.7ex\hbox{${\text{c}}$} \!\mathord{\left/ {\vphantom {{\text{c}} {\text{t}}}}\right.\kern-0pt} \!\lower0.7ex\hbox{${\text{t}}$}}}} }} } & {\dot{\varepsilon }_{{\text{p}}}^{{{\raise0.7ex\hbox{${\text{c}}$} \!\mathord{\left/ {\vphantom {{\text{c}} {\text{t}}}}\right.\kern-0pt} \!\lower0.7ex\hbox{${\text{t}}$}}}} { > }\dot{\varepsilon }_{{\text{p}}} } \\ {{\upgamma }_{{{\raise0.7ex\hbox{${\text{c}}$} \!\mathord{\left/ {\vphantom {{\text{c}} {\text{t}}}}\right.\kern-0pt} \!\lower0.7ex\hbox{${\text{t}}$}}}} \sqrt {\dot{\varepsilon }_{{\text{p}}} } } & {\dot{\varepsilon }_{{\text{p}}} { > }\dot{\varepsilon }_{{\text{p}}}^{{{\raise0.7ex\hbox{${\text{c}}$} \!\mathord{\left/ {\vphantom {{\text{c}} {\text{t}}}}\right.\kern-0pt} \!\lower0.7ex\hbox{${\text{t}}$}}}} } \\ \end{array} } \right.$$

The parameters involved in the expression is shown in Eq. (22).

22 $$\beta_{{\text{c}}} {\text{ = 4/(20 + 3f}}_{{\text{c}}} {), }\beta_{t} = 2/\left( {20 + f_{c} } \right)$$

According to the continuity requirements, input the rate parameters through. The plastic strength factor is shown in Eq. (23).

23 $${{F}}_{{{e}}} \left( {{{p}}^{*} } \right){ = }\left\{ {\begin{array}{*{20}c} {{{g}}_{{{c}}}^{*} } & {{{3p}}^{*} \ge {{F}}_{{{r}}}^{{{c}}} {{g}}_{{{c}}}^{*} } \\ {{{g}}_{{{c}}}^{*} - \frac{{{{3p}}^{*} - {{F}}_{{{r}}}^{{{c}}} {{g}}_{{{c}}}^{*} }}{{{{F}}_{{{r}}}^{{{c}}} {{g}}_{{{c}}}^{*} {{ + F}}_{{{r}}}^{{{t}}} {{g}}_{{{t}}}^{*} {{f}}_{{{t}}}^{*} }}\left( {{{g}}_{{{t}}}^{*} - {{g}}_{{{c}}}^{*} } \right)} & {{{F}}_{{{r}}}^{{{c}}} {{g}}_{{{c}}}^{*} > {{3p}}^{*} \ge - {{F}}_{{{r}}}^{{{t}}} {{g}}_{{{t}}}^{*} {\text{f}}_{{{t}}}^{*} } \\ {{{g}}_{{{t}}}^{*} } & { - {{F}}_{{{r}}}^{{{t}}} {{g}}_{{{t}}}^{*} {{f}}_{{{t}}}^{*} > {{3p}}^{*} } \\ \end{array} } \right.$$

Among that, ${p}_{c}^{*}={p}_{c}/{f}_{c}$, and ${p}_{u}^{*}={F}_{r}^{c}/{g}_{c}^{*}+{G}^{*}{\varepsilon }_{p}/{f}_{c}$. The hardening behavior is linear with the plastic strain.

24 $$\varepsilon_{{{p}}}^{*} {{ = min}}\left( {\frac{{\varepsilon_{{{P}}} }}{{\varepsilon_{{{p}}}^{{{h}}} }}{,1}} \right)$$

25 $$\varepsilon_{{{p}}}^{{{h}}} { = }\frac{{\sigma_{{{y}}} \left( {{{p}}^{*} {,}\;{{s,}}\;\dot{\varepsilon }_{{{p}}} {,}\;\varepsilon_{{{p}}}^{*} } \right)\left( {{1} - {{F}}_{{{e}}} {{F}}_{{{c}}} } \right)}}{{\gamma {{3G}}^{*} }}$$

26 $${{G}}^{*} { = }\xi {{G}}$$

Among that, *G*-shear modulus of the original material, $\xi$-hardening attenuation coefficient in the model. When the hardening state reaches the ultimate strength of the failure surface, the damage further accumulates in the plastic strain and inelastic loading process. The failure elastic strain equation is shown as (27)

27 $$\varepsilon_{{\text{p}}}^{{\text{f}}} { = }\left\{ {\begin{array}{*{20}c} {{\text{D}}_{{1}} \left[ {{\text{p}}^{*} - \left( {\text{1 - D}} \right){\text{p}}_{{\text{t}}}^{*} } \right]^{{{\text{D}}_{{2}} }} } & {{\text{p}}^{*} \ge \left( {{1} - {\text{D}}} \right){\text{p}}_{{\text{t}}}^{*} { + }\left( {\frac{{\varepsilon_{{\text{p}}}^{{\text{m}}} }}{{{\text{D}}_{{1}} }}} \right)^{{{\raise0.7ex\hbox{${1}$} \!\mathord{\left/ {\vphantom {{1} {{\text{D}}_{{2}} }}}\right.\kern-0pt} \!\lower0.7ex\hbox{${{\text{D}}_{{2}} }$}}}} } \\ {\varepsilon_{{\text{p}}}^{{\text{m}}} } & {\left( {{1} - {\text{D}}} \right){\text{p}}_{{\text{t}}}^{*} { + }\left( {\frac{{\varepsilon_{{\text{p}}}^{{\text{m}}} }}{{{\text{D}}_{{1}} }}} \right)^{{{\raise0.7ex\hbox{${1}$} \!\mathord{\left/ {\vphantom {{1} {{\text{D}}_{{2}} }}}\right.\kern-0pt} \!\lower0.7ex\hbox{${{\text{D}}_{{2}} }$}}}} > {\text{p}}^{*} } \\ \end{array} } \right.$$

The damage parameters are accumulated by plastic strain, shown as (28).

28 $${{D = }}\mathop \smallint \limits_{{\varepsilon_{{{p}}}^{{{h}}} }}^{{\varepsilon_{{{p}}} }} \frac{{{{d}}\varepsilon_{{{p}}} }}{{\varepsilon_{{{p}}}^{{{f}}} }}$$

The damage surface equation is shown as Eq. (28).

29 $$\sigma_{{{d}}} \left( {{{p}}^{*} {,}\;{{s,}}\;\dot{\varepsilon }_{{{p}}} } \right){ = }\left\{ {\begin{array}{*{20}c} {\sigma_{{{y}}} \left( {{{p}}^{*} {,}\,{{s,}}\,\dot{\varepsilon }_{{{p}}} {,1}} \right)\left( {{1} - {{D}}} \right){{ + Df}}_{{{c}}} \sigma_{{{r}}}^{*} {{p}}^{*} } & {{0} \le {{p}}^{*} } \\ {\sigma_{{{y}}} \left( {{{p}}^{*} {,}\;{{s,}}\;\dot{\varepsilon }_{{{p}}} {, 1}} \right)\left( {{1} - {{D}} - \frac{{{{p}}^{*} }}{{{{p}}_{{{t}}}^{*} }}} \right)} & {\left( {{1} - {{D}}} \right){{p}}_{{{t}}}^{*} \le {{p}}^{*} < {0}} \\ \end{array} } \right.$$

Among that, $\sigma_{{\text{r}}}^{*} \left( {{\text{p}}^{*} } \right){\text{ = A}}_{{\text{f}}} \left( {{\text{p}}^{*} } \right)^{{{\text{n}}_{{\text{f}}} }}$, plastic flow along the direction of deviatoric stress ${\dot{\varepsilon }}_{p}\sim s$. However, for tension, the parameter PFC can be selected to correspond to the influence value of plastic volumetric strain, $\lambda \le 1$ is used to indicate the variable, the special value $\lambda = 1$, $\dot{\varepsilon }_{{\text{p}}} \sim {\text{s}} - {\text{pI}}$.

### Established and modified model

#### Established the numerical simulation model

**Figure 2:**
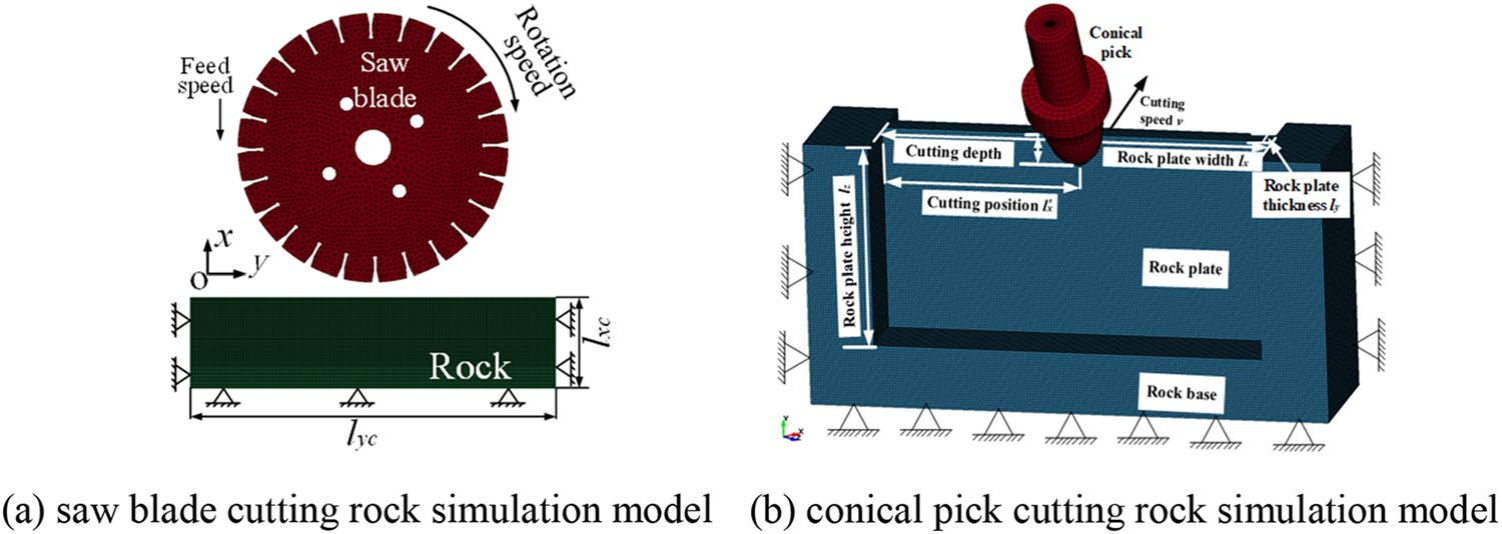
The numerical simulation of combined rock breaking with saw blade and conical pick.

According to combined rock breaking method with saw blade and conical pick in the actual work progress, establish the saw blade cutting rock simulation model and conical pick cutting rock plate simulation model. The structural model of SolidWorks was imported into ANSYS/LS-DYNA to establish the simulation model. The structural model is meshed with the ANSYS/MeshTool with SOLID_164. The saw blade and conical pick material are defined as MAT_RIGID, and the rock material is defined as RHT, and rock key parameters is shown in Table 1. The contact type between the cutting tool and rock is defined as ERODING_SURFACE_TO_SURFACE. The constraints were added to the rock, the full constraints is added to the bottom surface of rock, the x-axial and y-axial displacement constraints is added to the right and left surface of rock, and the y-axial and z-axial displacement constraints is added to the front and behind the surface of the rock. The loads are added to the cutting tool, the rotary load around the x-axial, displacement along y-axial and z-axial added to the saw blade. The displacement load along x-axial added to the conical pick. The numerical simulation model of saw blade cutting rock and conical pick cutting rock are shown in Fig. 2. The calculation step is set to 0.01 s and the cutting speed and cutting distance define the calculation. After the setting are completed, the K file generated and imported into ANSYS/SLOVER for a solution. The high-performance WorkStation with 40 computing cores and 128 GB memory is used.

**Table 1:** The key parameters of rock material.

Parameter	Density (kg/m^3^)	Compressive strength (MPa)	Tensile strength (MPa)	Poission’s ratio
Value	2578.26	151.30	6.79	0.208

#### Quantitative analysis system of rock damage

**Figure 3:**
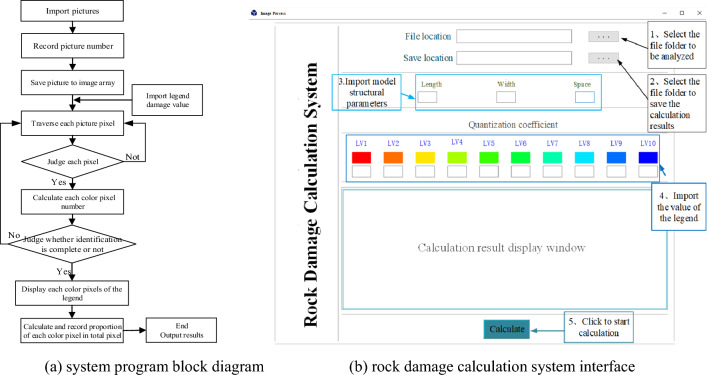
The rock damage calculation system.

The quantitative analysis model of rock damage based on image recognition is established, and the quantitative analysis software of rock damage based on Python is compiled to quantify the rock damage and realize the quantitative analysis of rock damage. Based on ANSYS/LS-DYNA, the combined rock breaking of saw blade and pick was numerically simulated. Use LS-PrePost to post process the numerical simulation results and slice the rock damage model at a specific interval to form a rock damage picture. The rock damage pictures are imported into the rock damage calculation system. The system analyzes each damage picture separately and then quantifies the rock damage volume with the cumulative method. The block diagram of the rock damage analysis software is shown in Fig. 3a. Quantitative analysis software for rock damage is established and packaged based on Python to simplify the steps of analyzing rock damage, as shown in Fig. 3b. Import the storage path of the rock damage slice image into the software, set the output position, rock size and damage scale value, and click the calculation to realize the quantitative processing of rock damage.

#### Verification and modification

**Figure 4:**
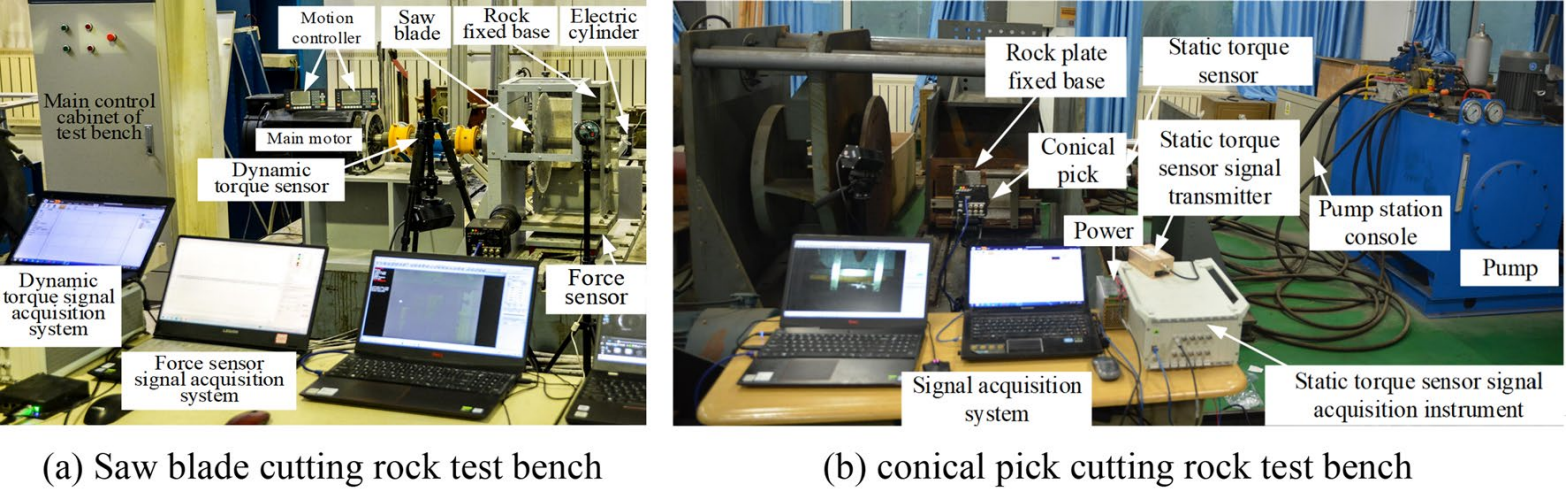
The experimental bench of combined rock breaking and traditional rock breaking.

Built a rock breaking experiment system of combined rock breaking with saw blade and conical pick and a tradition rock breaking experiment system, as shown in Fig. 4. Conduct rock breaking experiments and verify the numerical simulation model. The combined rock breaking with saw blade and conical pick multi-function test bench can complete the saw blade cutting experiment and the conical pick cutting rock with saw blade pre-cutting experiment. The saw blade rock cutting test bench mainly includes the main control cabinet, servo motor, electric cylinder, servo control system, rock fixed base and signal acquisition system. The rock pre-cutting test bench for pick cutting saw blade mainly includes: rock fixed base, cutting components, static torque sensor, pump station, pump station control system, signal acquisition system, etc.

**Figure 5:**
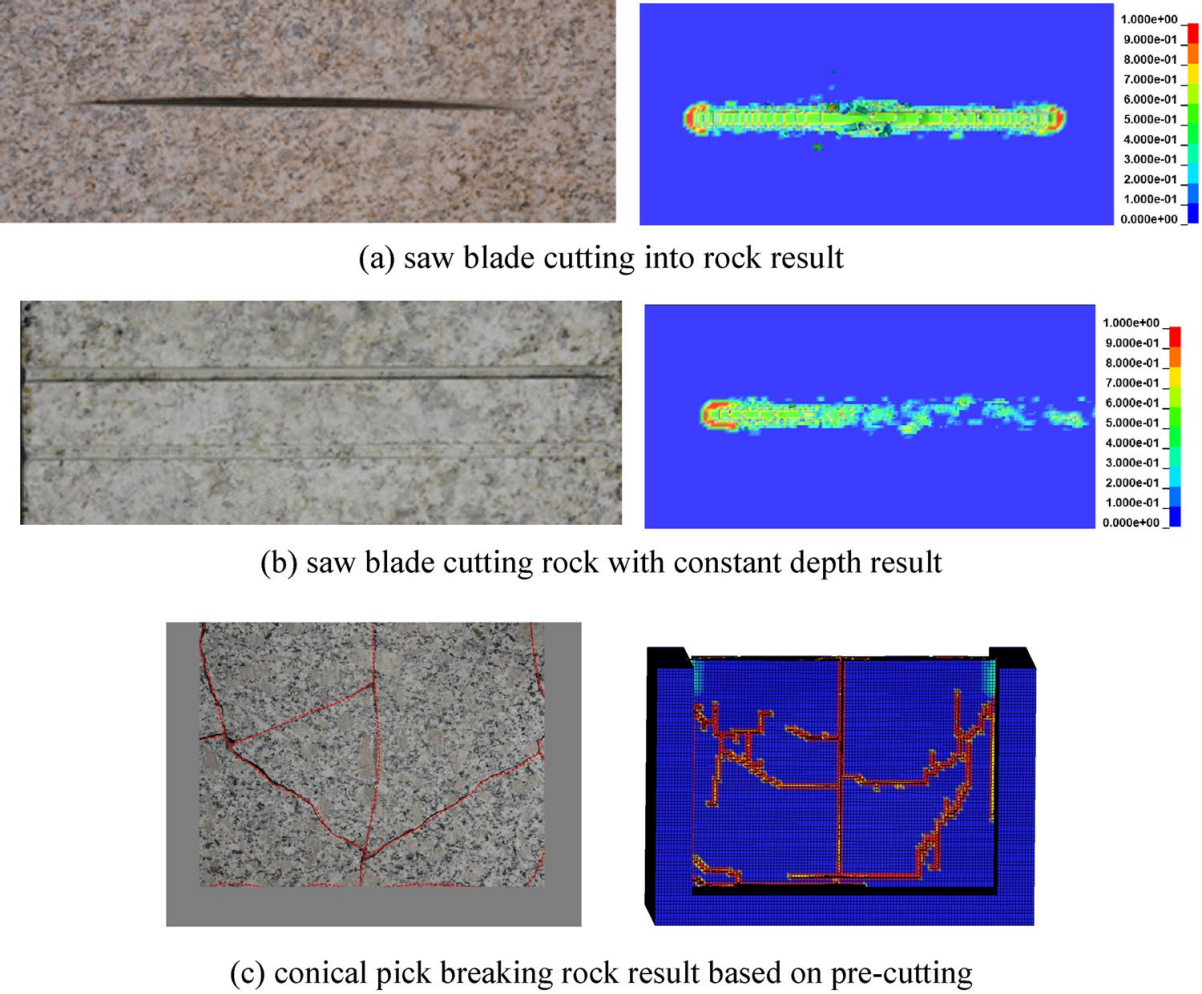
The results of combined rock breaking with saw blade and conical pick.

Saw blade cuts into rock with the rotation speed of 1000 r/min, a feed speed of 0.20 m/min, and the experiment and numerical simulation result is shown as Fig. 5a. The result of a saw blade cutting rock with constant experiment and numerical simulation with a rotation speed of 1000 r/min, feed speed of 0.20 m/min, and cutting depth of 5 mm is shown as Fig. 5b. The result of experiment and numerical simulation with a cutting speed of 1 m/s, and cutting angle of 50°, cutting depth of 15 mm, cutting rock plate with a width of 300 mm, the height of 150 mm and a thickness of 20 mm is shown as Fig. 5c. A comparison of the experiment and numerical simulation results can be indicated that the saw blade cuts into the rock to form a saw joint, which is basically the same. The rock walls on both sides of the middle position of the saw joint are broken to a certain extent. The saw blade cuts the rock with a constant cutting depth to form a saw joint, forming a certain crushing range on the rock walls on both sides of the saw joint, as shown in Fig. 5b. According to the numerical simulation results of the rock cut by the conical pick with pre-cutting, it can be clearly seen that the rock plate fracture of the rock with pre-cutting by the conical pick is similar, and broken volume is basically the same, as shown in Fig. 5c.

**Figure 6:**
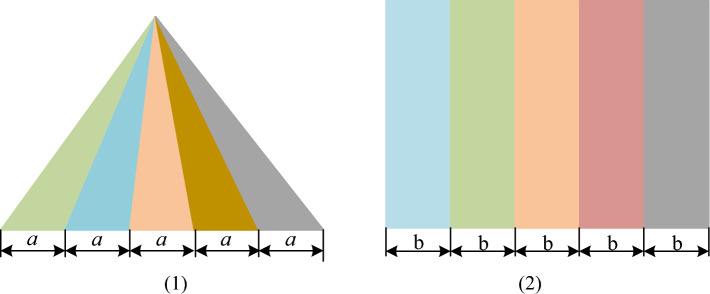
The test pictures verified rock damage calculation system.

In order to verified the quantitative analysis system of rock damage, the test pictures are imported into the system. The test pictures are shown in Fig. 6, there are five different color area with same size in each picture.

The test result of rock damage calculation system analysis the test pictures is shown in the Table 2. There are five colors of the same proportion and size in the test picture. Therefore, it is obvious that the analysis result of test pictures is accuracy. The test picture result error is 0.003 and 0.002, respectively, which is less than 0.005, shown as Table 2. The rock damage calculation system is accurate.

**Table 2:** The test result of rock damage calculation system analysis test pictures.

	Proportion of each color area					Error
Picture 1	0.201	0.199	0.203	0.200	0.197	0.003
Picture 2	0.198	0.202	0.200	0.199	0.201	0.002

## Results and discussion

### Combined rock breaking progress

**Figure 7:**
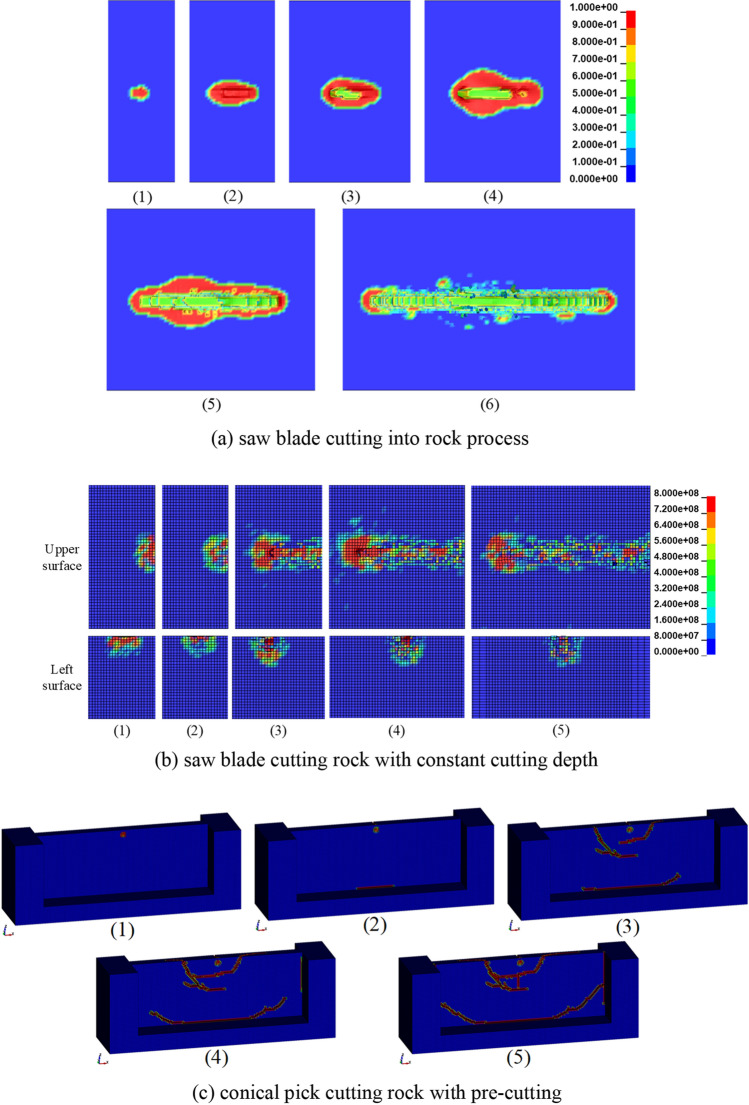
The process of combined rock breaking with saw blade and conical pick.

According to the combined rock breaking method of saw blade and conical pick in engineering application, the rock is first cut by the saw blade and then cut by conical pick. The saw blade cutting rock progress can be divided into saw blade cutting into rock progress and cutting rock with constant cutting depth. The result of saw blade cuts into rock with rotation speed of 1500 r/min, feed speed of 0.30 m/min is shown as Fig. 7a. And result of saw blade cutting rock with rotational speed of 1500 r/min, feed speed of 0.30 m/min and cutting depth of 30 mm is shown as Fig. 7b. The process of conical pick cutting rock with cutting speed of 2.0 m/s, cutting depth of 20 mm and cutting angle of 50°, and the structural parameters of the rock with width of 300 mm, height of 200 mm and thickness of 30 mm is shown as Fig. 7c.

The process of saw blade cutting into rock is shown as Fig. 7a. The saw blade cuts into rock with stable rotation speed and feed speed. When the saw blade contacts the rock, the rock damage appears at the contact point between the saw blade and rock, shown as Fig. 7a (1). With saw blade continue cutting rock, the rock element appear deformation with the shear action, shown as Fig. 7a (2). With the cutting distance of saw blade increasing, some rock elements are failing and deleting, forming joint formation, shown as Fig. 7a (4). A certain width of damage area is formed on both sides of the saw seam, the position with the widest damage width has a certain distance offset along the direction of saw blade rotation, as shown in Fig. 7a (5). The saw blade continues to cut into the rock, and the damage scope on both sides of the saw joint gradually narrows, and the rock damage scope is mainly concentrated at the front and rear ends of the saw blade, as shown in Fig. 7a (6).

The process of rock cutting by saw blade at fixed depth is shown in Fig. 7b. The saw blade cuts the rock at a stable feed speed, rotation speed and cutting depth. When the saw blade touches the rock, the rock damage occurs at the contact point between the saw blade and the rock, as shown in Fig. 7b (1). As the saw blade continues to cut the rock, the range of rock damage gradually increases, and the saw blade cuts the stable width and depth of the saw seam formed by the rock. The rock pre-cutting process of the pick cutting saw blade is shown in Fig. 7c. The pick contacts the plate rock, and the contact point between the pick and the rock plate is damaged. The pick cuts the rock plate with stable cutting parameters, and the rock plate begins to crack. With the increase of cutting distance, the crack continues to expand, and the rock plate intersects with the crack breaks, as shown in Fig. 7c.

### Impact of sawing on rock damage

#### The sawing into rock process

In the saw blade cutting into rock, the cutting parameters (rotation speed and feed speed) greatly influence rock damage. The saw blade cuts rock with a rotation speed of 1000 r/min, 1600 r/min, 2200 r/min, and 2800 r/min, feed speeds of 0.10 m/min, 0.16 m/min, 0.22 m/min, and 0.28 m/min, to study the influence of cutting parameter on rock damage.

**Figure 8:**
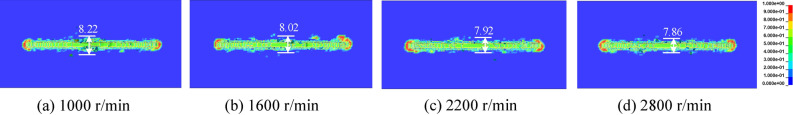
The rock damage cloud with different rotation speeds.

The rock damage with different rotation speeds is shown in Fig. 8. The saw blade cuts rock with a feed speed of 0.20 m/min, and a rotation speed of 1000 r/min, 1600 r/min, 2200 r/min and 2800 r/min. According to the rock damage cloud with the same cutting speed and various rotation speeds, the rock damage area declines with the rotation speed increasing at the same cutting depth.

**Figure 9:**
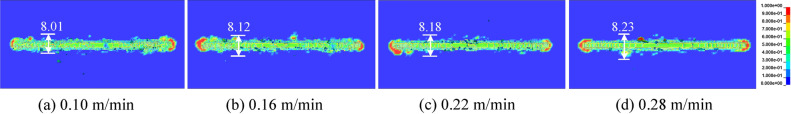
The rock damage cloud with different feed speeds.

The saw blade cuts rock with rotation speed of 2200 r/min, feed speed of 0.10 m/min, 0.16 m/min, 0.22 m/min and 0.28 m/min, the rock damage cloud with different feed speed is shown as Fig. 9. According to the rock damage cloud with same rotation speed and different feed speed, it is obvious that the rock damage area increase with feed speed increasing at the cutting depth.

**Figure 10:**
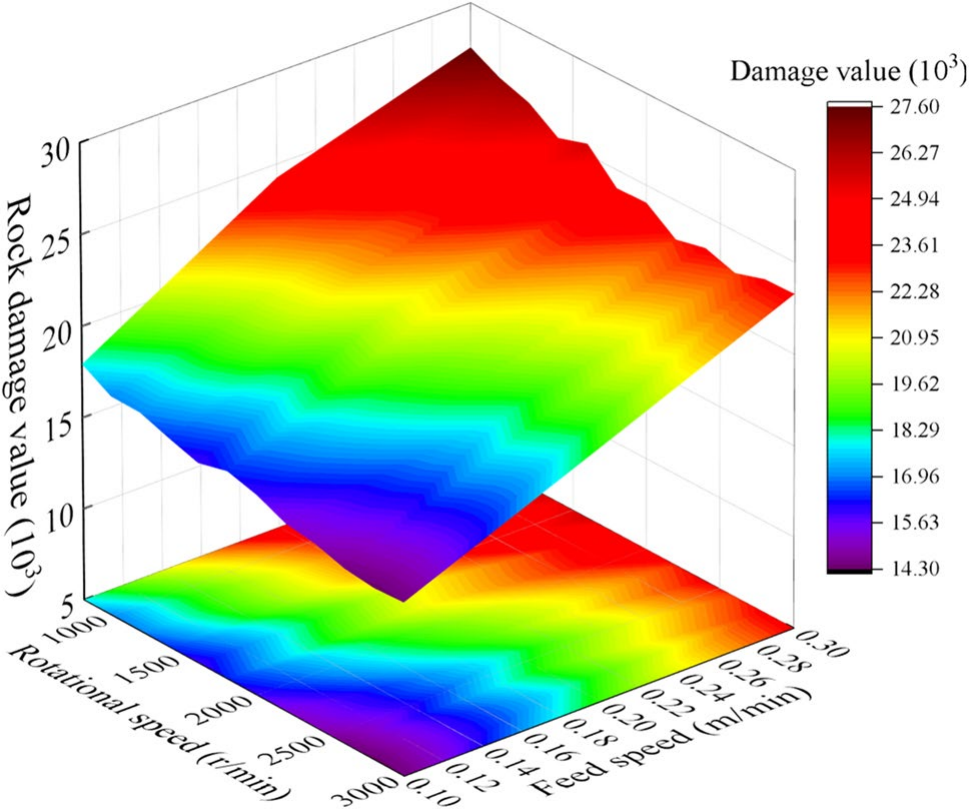
The rock damage cloud with different cutting parameters.

Based on the quantitative analysis of rock damage by the statistical program of rock damage, the influence of saw blade rotation speed on rock damage is studied by the method of quantitative analysis. The relationship between rock damage and saw blade cutting parameters is shown in Fig. 10. The rock damage value increases with feed speed increasing and rotation speed declining.

#### The sawing rock with constant depth process

The process of saw blade cutting rock with constant depth is the main part of rock cutting process by saw blade. The effects of cutting parameters such as saw blade rotation speed, feed speed and cutting depth on rock damage are studied. The saw blade cuts rock with constant depth, with rotation speed of 1000 r/min, 1500 r/min, 2000 r/min, 2500 r/min and 3000 r/min, feed speed of 0.10 m/min, 0.15 m/min, 0.20 m/min, 0.25 m/min and 0.30 m/min and cutting depth of 4 mm, 8 mm, 12 mm, 16 mm and 20 mm. To investigate the cutting parameters of saw blade effect on rock damage.

**Figure 11:**
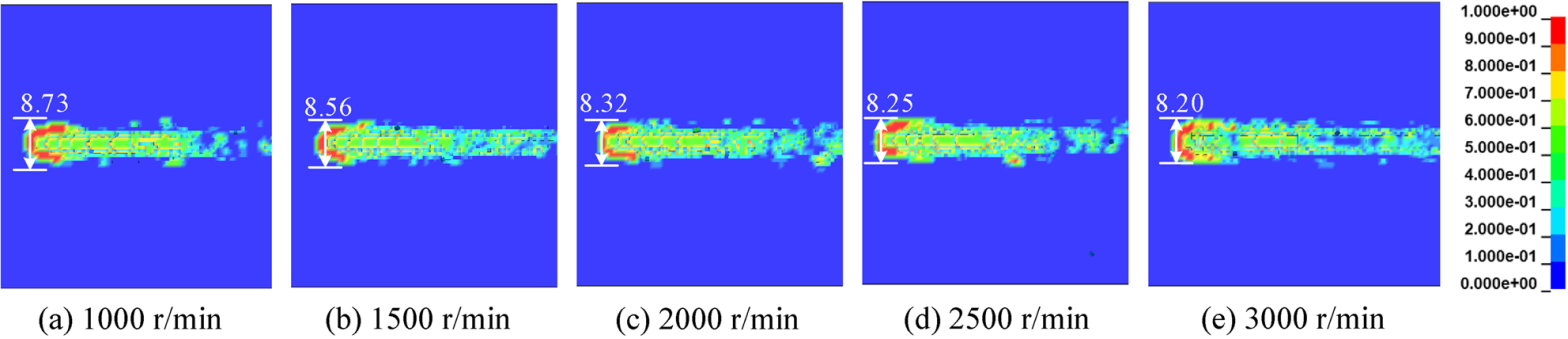
The rock damage cloud with different rotation speeds.

The saw blade cuts rock with constant depth at feed speed of 0.30 m/min, cutting depth of 20 mm rotation speed 1000 r/min, 1500 r/min, 2000 r/min, 2500 r/min and 3000 r/min. The result of saw blade cutting rock with different rotation speed is shown as Fig. 11. According to the saw blade cutting rock with different rotation speed results, it can be seen that the rock damage are decreasing with the rotation speed increasing.

**Figure 12:**
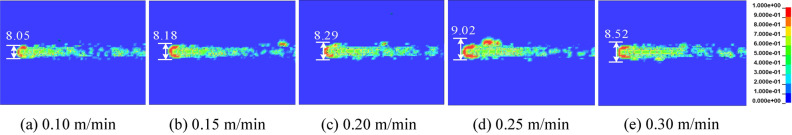
The rock damage cloud with different feed speeds.

The saw blade cuts rock with cutting depth of 20 mm, rotation speed of 2000 r/min and feed speed of 0.10 m/min, 0.15 m/min, 0.20 m/min, 0.25 m/min and 0.30 m/min. The result of saw blade cutting rock with different feed speeds is shown in Fig. 12. Compared the rock damage cloud with different feed speeds, the rock damage area increasing with the feed speed increasing. The feed speed of saw blade have great effect on rock damage.

**Figure 13:**
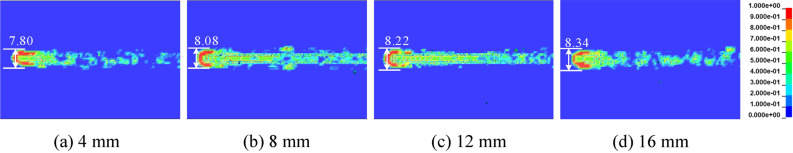
The rock damage cloud with different cutting depths.

The saw blade cuts rock with rotation speed of 2000 r/min, feed speed of 0.20 m/min and cutting depth of 4 mm, 8 mm, 12 mm, 16 mm and 20 mm. The saw blade cutting rock with different cutting depth result is shown in Fig. 13. Compared with the rock damage cloud with different cutting depth, the rock damage area of rock increases with the saw blade cutting depth increasing.

**Figure 14:**
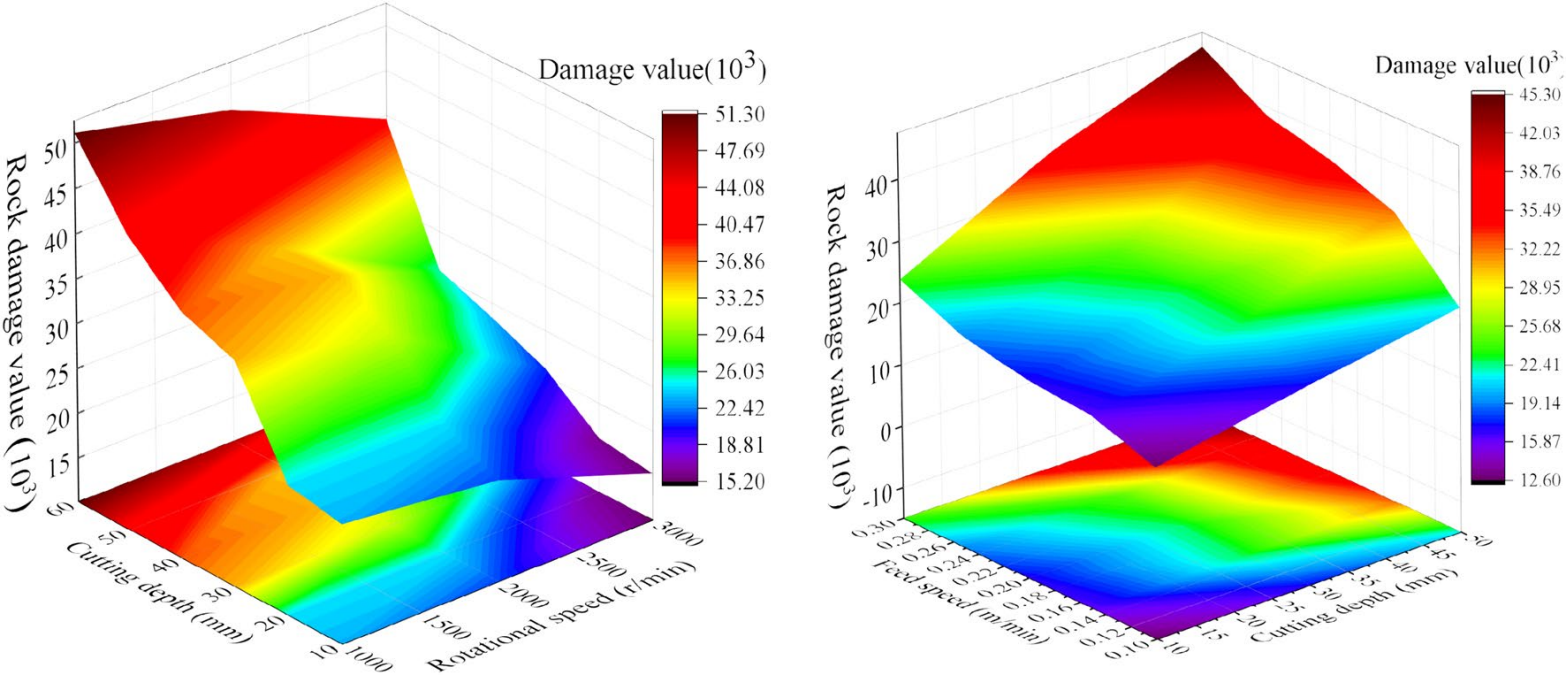
The rock damage cloud with different cutting parameters.

The rock damage value has been influenced by the saw blade cutting parameters. The rock damage value with different cutting parameters is shown in Fig. 14. The result indicated that the rock damage value increases with feed speed and cutting depth increasing and rotation speed declining. And the changing trends of the rock damage area is similar with the research result of Wang et al.^40, 41^.

### The impact factor of rock damage

The process of combined rock breaking with saw blade and conical pick, conical pick breaking rock with saw blade pre-cutting is the essential process of the combined rock breaking. The conical pick cutting rock plate is influenced by the cutting parameters and structural parameters. The conical pick with feed speed of 0.50 m/s, 1.0 m/s, 1.5 m/s, 2.0 m/s, 2.5 m/s and 3.0 m/s, the cutting depth of 5 mm, 10 mm, 15 mm, 20 mm, 25 mm and 30 mm, and the cutting angle of 30°, 40°, 45°, 50°, 60° and 70° and the cutting station (${l}_{x}{\prime}/{l}_{x})$ of 1/10, 1/5, 3/10, 2/5 and 1/2. And the rock plate with width of 50 mm, 70 mm, 90 mm, 150 mm, 200 mm, 300 mm and 500 mm, height of 50 mm, 75 mm, 100 mm, 150 mm, 200 mm, 300 mm and 400 mm, thickness of 5 mm, 10 mm, 15 mm, 20 mm, 25 mm and 30 mm, to study the cutting parameters and structural parameters on rock damage.

**Figure 15:**
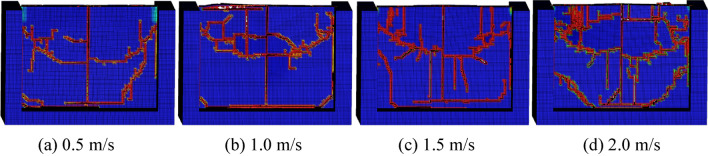
The rock damage cloud with different cutting speeds.

The conical pick cuts rock with feed speed of 0.50 m/s, 1.0 m/s, 1.5 m/s and 2.0 m/s, cutting angle of 50°, cutting depth of 30 mm and the cutting position of 1/2, and rock plate structural parameters with width of 300 mm, height of 200 mm and thickness of 20 mm, to study the feed speed on rock damage. The result of conical pick cutting rock plate with different feed speeds is shown as Fig. 15. The feed speed increasing causes the rock plate creaks and rock fragments number increasing.

**Figure 16:**
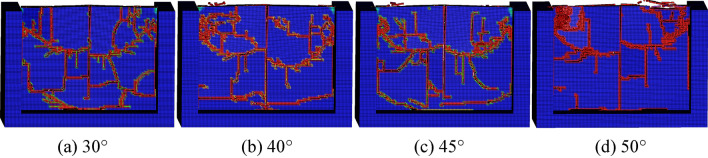
The rock damage cloud with different cutting angles.

The conical pick cutting rock plate with cutting angle of 30°, 40°, 45° and 50°, to research the cutting angle effect on rock plate breaking, the numerical simulation results are shown as Fig. 16. The numerical simulation result of conical pick cutting rock plate with different cutting angle indicated that the rock cracks and rock fragments increases and then declines with cutting angle increasing.

**Figure 17:**
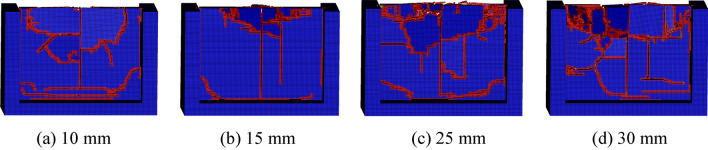
The rock damage cloud with different cutting depths.

The conical pick cuts rock plate with different cutting depth to investigate the effect of cutting depth on rock breaking. The conical pick cuts rock plate with the cutting depth of 10 mm, 15 mm, 25 mm and 30 mm, and result of conical pick cutting rock is shown in the Fig. 17. The result of rock breaking indicated that the number of crack and rock fragment increases with cutting depth increasing. The rock plate is basically stripped from the base rock. Therefore, the fragmentation of rock plate can adjusted by controlling the cutting depth.

**Figure 18:**
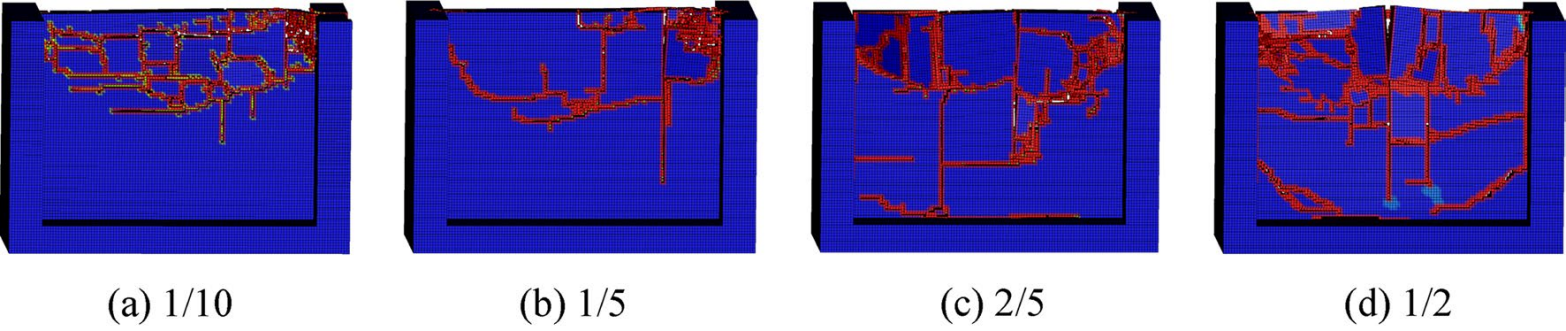
The rock damage cloud with different cutting positions.

The conical pick cutting rock plate with cutting position of 1/10, 1/5, 3/10, 2/5 and 1/2 to investigate the effect of cutting position on rock plate breaking, the numerical simulation results are shown in Fig. 18. Compared with conical pick cutting rock plate of different cutting position, with the cutting position close to the middle position of rock plate, the rock breaking volume increases. Therefore, the cutting position of conical pick cutting rock have great influence on rock breaking.

**Figure 19:**
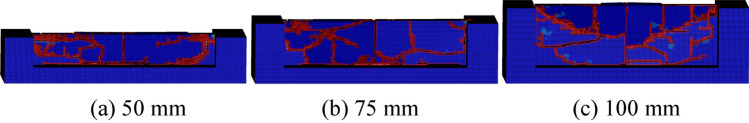
The rock damage cloud of rock plate with different height.

The rock plate structural parameters have great influence on rock breaking with the conical pick. The rock plate structural parameters include height, width and thickness. Therefore, the height of rock plate has been set as 50 mm, 75 mm, 100 mm, 150 mm, 200 mm, 300 mm and 400 m to study the rock plate height influence on rock breaking. The result of conical pick cutting rock plate with different is shown in Fig. 19. It is obvious that the rock plate breaking ideal with the height increasing. However, the rock plate with different height is crushing completely.

**Figure 20:**
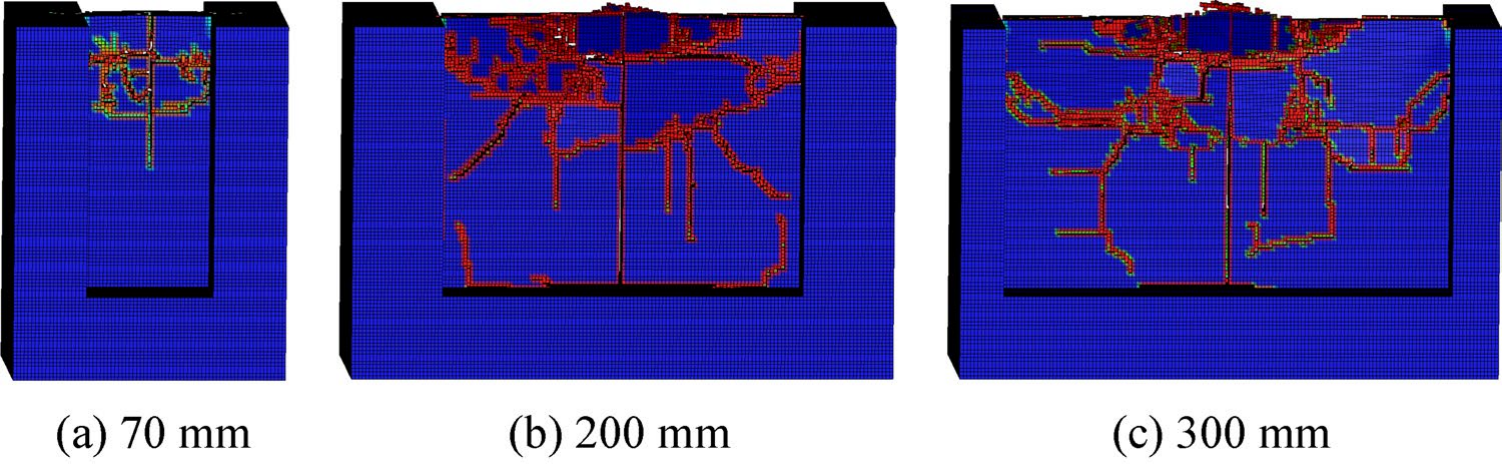
The rock damage cloud of rock plate with different width.

The conical pick cutting rock plate with width of 50 mm, 70 mm, 90 mm, 150 mm, 200 mm, 300 mm and 500 mm to investigate the width of rock plate influence on rock breaking. The width of rock plate has great influence on rock breaking, according to the rock plate. The rock plate breaking volume, rock cracks and fragments number increases with the width of rock plate increasing is show as Fig. 20.

**Figure 21:**
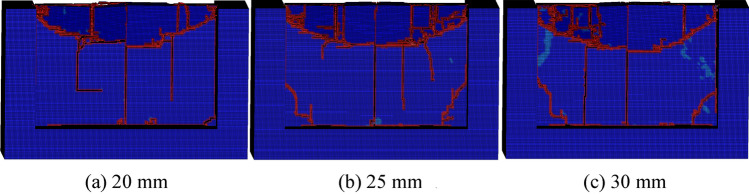
The rock damage cloud of rock plate with different thickness.

The thickness of rock plate is an important parameter of the rock plate. Therefore, the thickness of rock plate has been researched, by the thickness of rock plate setting as 5 mm, 10 mm, 15 mm, 20 mm, 25 mm and 30 mm. The results of conical pick cutting rock plate with various thickness is shown as Fig. 21. According to the result of conical pick cutting rock plate, the thickness of rock plate has great effect on rock breaking. The rock plate breaking volume increases with the thickness of rock plate increasing.

**Figure 22:**
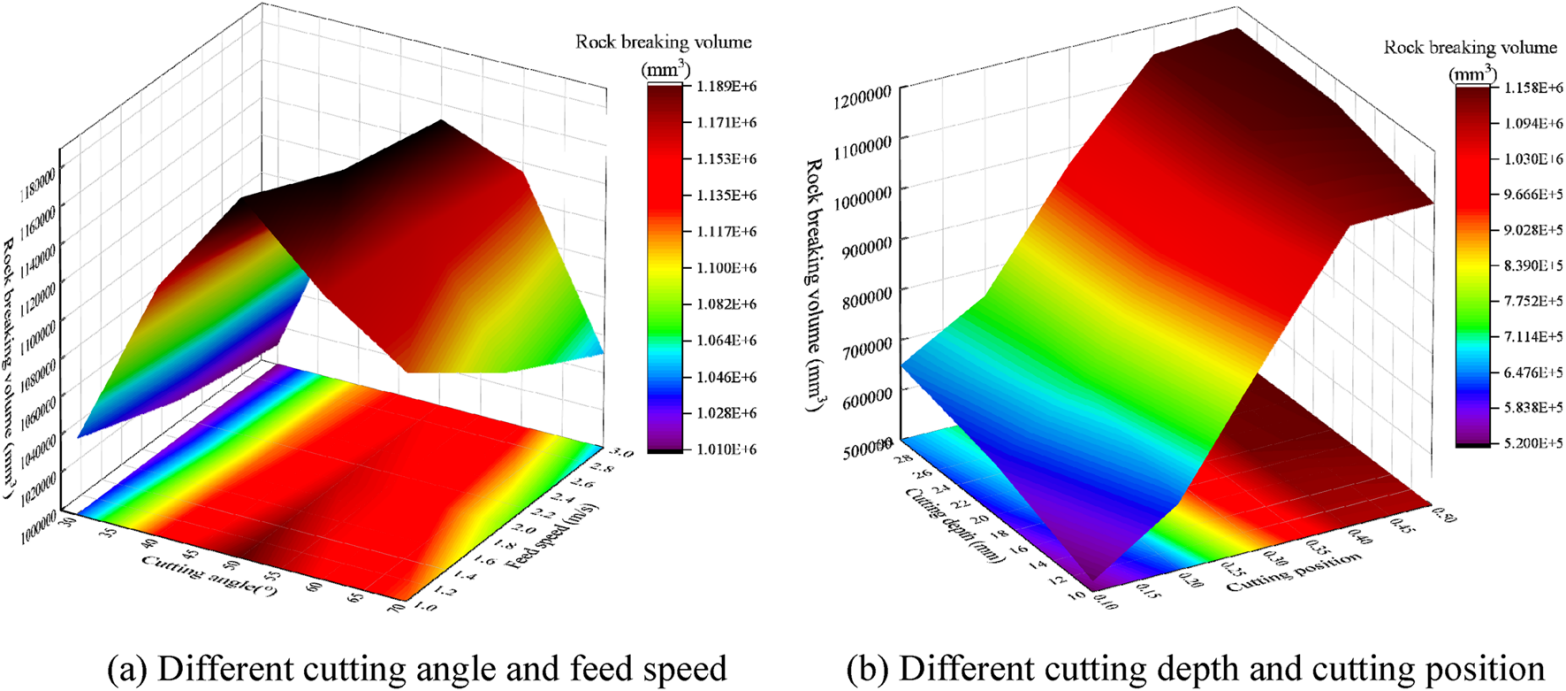
The rock breaking volume with various cutting parameters.

**Figure 23:**
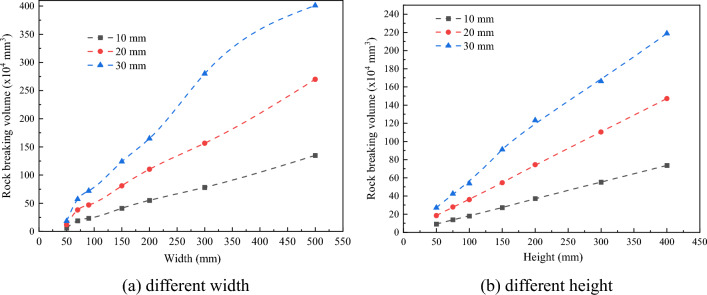
Rock damage value with different cutting parameters and rock plate structural parameters.

The cutting parameters of conical pick and the structural parameters of rock plate have great influence on conical pick cutting rock plate. The cutting parameters and structural parameters have influence on rock breaking volume and the rock crack number, as shown in Fig. 22 and Fig. 23. The rock breaking volume increases with the cutting depth and cutting position increasing and feed speed decreasing. And the rock breaking volume of conical pick cutting rock plate first increases and then decrease with the cutting angle increasing.

The rock plate structural parameters have great influence on rock breaking volume. The rock breaking volume of conical pick cutting rock plate increases with the width, height and thickness of rock plate increasing. The rock breaking volume is linear with width, height and thickness of rock plate, as shown in Fig. 23. The research results indicated that increasing the rock plate width and height cloud increase the rock plate breaking efficiency.

## Conclusions

The paper establishes the numerical simulation model of combined rock breaking with saw blade and conical pick based on ANSYS/LS-DYNA to research cutting parameters and rock structural parameters on rock damage.

1. The numerical simulation model of combined rock breaking with saw blade and conical pick has been established, and numerical simulation model is verified and modified with the experiment of saw blade and conical pick cutting rock.
2. The rock damage in the process of saw blade cutting rock increases with the feed speed and cutting depth increasing, and the rotation speed increasing causes the rock damage declining.
3. The conical pick cuts rock plate with different structural parameters, the structural parameters have great influence on rock, the width, height and thickness of rock plate and feed speed, cutting angle and cutting position of conical pick affect the rock breaking capacity of the conical pick.

The investigation results can be applied to optimize cutting parameters of saw blade and cutting parameters of conical pick to improve the combined rock breaking efficiency.

## Data Availability

The data used to support the findings of this study are included within the article.
